# Increasing radiosensitivity with the downregulation of cofilin-1 in U251 human glioma cells

**DOI:** 10.3892/mmr.2014.3125

**Published:** 2014-12-22

**Authors:** HUA-QING DU, LING CHEN, YING WANG, LI-JUN WANG, HUA YAN, HONG-YI LIU, HONG XIAO

**Affiliations:** 1 Neuro-Psychiatric Institute, Nanjing Medical University, Affiliated Nanjing Brain Hospital, Nanjing, Jiangsu 210029, P.R. China; 2 Department of Neurosurgery, Nanjing Medical University, Affiliated Nanjing Brain Hospital, Nanjing, Jiangsu 210029, P.R. China

**Keywords:** glioma, human U251 cells, cofilin-1, radiosensitivity

## Abstract

The aim of the present study was to examine the association between cofilin-1 (CFL1) and radioresistance in human glioma U251 cells. CFL1 expression was downregulated and upregulated in U251 cells through the transfection of CFL1-small interfering (si)RNA and pcDNA3.1-CFL1, respectively. The radiosensitivity of U251 cells and established radioresistant U251 cells (RR-U251) was evaluated using cell viability, migration and invasion ability assays. Cell cycle distribution was also examined. The results showed that CFL1 expression was significantly increased in RR-U251 cells; in addition, the cell viability, migration and invasion ability of RR-U251 cells were significantly enhanced compared to those of the normal U251 cells, whereas the number of cells arrested in G2 phase was markedly decreased. In CFL1-silenced RR-U251 and CFL1-silenced U251 cells, the cell viability, migration and invasion abilities were significantly downregulated and the number of cells arrested in G2 phase was increased compared to that of the untransfected cells. In U251 cells overexpressing CFL1, cell viability, migration and invasion abilities were markedly upregulated and the number of cells arrested in G2 phase was decreased. In conclusion, the results of the present study suggested that downregulation of CFL1 may increase radiosensitivity in U251 cells.

## Introduction

Glioma is the most prevalent type of brain tumor, accounting for >50% of all brain tumors (1), with one of the highest mortality rates of all cancers. Gliomas have a highly vascularized phenotype and at present, the primary treatment method is surgical therapy; however, complete surgical resection is difficult due to the infiltrative growth and invasiveness of gliomas. Radiotherapy has been used in combination with surgical therapy approaches; however, despite the successful development of novel radiotherapeutic strategies for the treatment of glioma, radioresistance remains a dominant and unresolved problem.

A previous study revealed that cofilin-1 (CFL1) was significantly upregulated in radioresistant astrocytomas (2), which indicated that CFL1 may be involved in the radioresistant phenotype and therefore may be a target for increasing radiosensitivity.

Cofilin genes have two subtypes which encode different proteins in mammals. CFL1, a member of the actin-depolymerizing factor family, is a small (19 kDa), ubiquitous cytoskeletal protein which is expressed in non-muscular cells, including nerve and liver cells (3). CFL1 is essential for the promotion of actin depolymerization/polymerization and the rapid turnover of actin filaments (4). Reorganization of the actin cytoskeleton is essential for tumor development as well as cell motility, adhesion, invasion and angiogenesis. A previous study demonstrated that CFL1 inhibition in carcinoma cells decreased cell motility (5); in addition, downregulation of cofilin reduced assembly and stability of the invadopodia, therefore indicating its critical role in cell invasion (6). Angiogenesis was found to be dependent on the CFL1-induced regulation of actin cytoskeletal dynamics; furthermore, CFL1 was reported to be the target of several angiogenesis inhibitors (7).

Apart from surgical resection, radiotherapy is the most effective method of glioma treatment; however, the major obstacle for effective radiotherapy is radioresistance. Previous studies have identified numerous factors which have been reported to influence the effectiveness of radiotherapy (8–13); however, to the best of our knowledge, there are no studies that have investigated an association between CFL1 and radiotherapy. The aim of the present study was to examine the potential association between CFL1 and radioresistance in human glioma cells.

## Materials and methods

### Cell culture

Human U251 cells, purchased from Nanjing KeyGEN Biotech Co., Ltd (Nanjing, China), were cultured in Dulbecco’s modified Eagle’s Medium (DMEM; Gibco-BRL, Carlsbad, CA, USA) supplemented with 10% fetal calf serum (Gibco-BRL). Cells were incubated at 37°C in 5% CO_2_ and routinely subcultured every day unless otherwise stated.

### Establishment of radioresistant U251 cells (RR-U251)

U251 cells were seeded at a density of 1×10^5^ in a T25 flask (Corning Inc., Corning, NY, USA) in complete medium. When cells reached 50% confluence they were treated with 5 Gy of radiation using a ^60^Co source (RuiDi Biotechnology, Nanjing, China) at 0.5 Gy/min. When cells reached 80% confluence, they were trypsinized (Trypsin; Sigma-Aldrich Shanghai Trading Co., Ltd, Shanghai, China) and subcultured into new flasks. When cells reached 50% confluence, the cells were serially irradiated with 5 Gy until 60 Gy of irradiation was reached, as previously described (14).

### Transfection

The sequences of CFL1-small interfering (si)RNA duplexes and the high expression plasmid pcDNA3.1-CFL1 were synthesized by GenePharma Co. Ltd (Shanghai, China). siRNA1 (5′-AGCGCAAGAAGGCGGUGCUTT-3′), siRNA2 (5′-GAGGAUCUGGUGUUUAUCUTT-3′) and siRNA3 (5′-GGUGUCAUCAAGGUGUUCATT-3′) were designed to target different coding regions of the human CFL1 messenger (m)RNA sequence (Gene ID, 1072).

U251 cells were seeded onto six-well plates in DMEM containing 10% fetal calf serum without penicillin or streptomycin and then incubated overnight. Cells were then transfected with CFL1-siRNAs or pcDNA3.1-CFL1 using Lipofectamin™ 2000 (Invitrogen Life Technologies, Carlsbad, CA, USA) according to the manufacturer’s instructions. Following 6 h of transfection, the medium was replaced with complete medium and the transfection efficiency was evaluated using a fluorescence microscope (Axiovert 40 CFL; Carl Zeiss, Oberkochen, Germany). CFL1 expression was analyzed at 24, 48 and 72 h following transfection. Cells transfected with pcDNA3.1-CFL1 were selected for stable clones using DMEM containing 400 μg/ml G418 (Sigma-Aldrich).

### Cell viability assay

Cell viability was determined using an MTT assay (Sigma-Aldrich, St Louis, MO, USA). Following radiotherapy, normal U251 cells, RR-U251 cells and the treated cells were seeded onto 96-well plates (5.0×10^3^ cells/well; n=6 for each condition). After 48 h, 20 μl MTT was added and incubated for 4 h prior to the addition of 150 μl DMSO. Then the optical density (OD) in each individual well was recorded at 570 nm using a microplate reader (Multiskan Ascent, model no. 354; Thermo Fisher Scientific, Shanghai, China). Cell viability was calculated as follows: Cell viability (100%) = (OD_treatment_/OD_control_)x100%.

### Cell migration assays

Wound healing assays were used to evaluate cell migration ability. Normal U251 and RR-U251 cells treated with CFL1-siRNA and pcDNA3.1-CFL1 were seeded onto six-well plates. Following radiotherapy, monolayers were disrupted to generate a linear wound using a 10-μl pipette tip. The six-well plates were washed twice with PBS and incubated with fresh medium. Images were captured at 0 and 24 h at identical sites using a fluorescence microscope, and the migration distance was measured. The migration ratio was calculated using the following formula: Migration ratio = [(Width_0 h_-Width_24 h_)/Width_0 h_] × 100%.

### Cell invasion assay

A cell invasion assay was performed using 24-well Transwell chambers (Corning, Inc.) and the inserts were coated with 50 μl Matrigel^®^ (Dilution, 1:8 with DMEM; BD Biosciences, Franklin Lakes, NJ, USA). Normal U251 and RR-U251 cells treated with CFL1-siRNA and pcDNA3.1-CFL1 were cultured in six-well plates. Following radiotherapy, the monolayer cells were trypsinized and transferred to the upper Matrigel chamber in 100 μl serum-free DMEM at a density of 1×10^5^/ml. DMEM supplemented with 15% fetal bovine serum (Gibco, Invitrogen Life Technologies) was added to the lower chamber as the chemoattractant. Following incubation for 24 h, cells remaining in the upper chamber were removed using cotton swabs, while invaded cells were fixed using dehydrated alcohol (Sigma-Aldrich), stained with crystal violet (Sigma-Aldrich) and then counted under a microscope (Axiovert 40 CFL). Images were captured in five randomly selected fields for each well (magnification, ×100). Three separate experiments were performed.

### Western blot analysis

Total protein was extracted from an equal number of cells in each group using radioimmunoprecipitation assay lysis buffer (Thermo Scientific, Waltham, MA, USA). Total protein (20 μg) was separated using 10% SDS-PAGE (Sunshine Biotechnology, Nanjing, China). The fractionated proteins were electro-transferred to a polyvinylidene fluoride membrane (Sunshine Biotechnology). The membrane was blocked in 5% skimmed milk (GuangMing, Nanjing, China) and probed with rabbit anti-CFL1 polyclonal primary antibodies (Abcam, Cambridge, MA, USA) diluted in Tris-buffered saline with Tween20 (1:500; Sunshine Biotechnology) overnight at 4°C. The membrane was then incubated with the appropriate horseradish peroxidase-conjugated polyclonal goat anti-rabbit secondary antibodies (1:10,000; Sunshine Biotechnology) for 2–3 h at room temperature. Immunoreactive bands were detected using a Supersignal west Pico Trial enhanced chemiluminescence kit (Thermo Fisher Scientific) and visualized using a Gel Image Analysis system (3400Mini; CLINX Science Instruments Co., Ltd, Shanghai, China).

### Reverse transcription quantitative polymerase chain reaction (RT-qPCR)

Total RNA was extracted from an equal number of cells in each group using the SV Total RNA Isolation System (Promega, Madison, WI, USA) according to the manufacturer’s instructions. Total RNA was then reverse transcribed to complementary DNA with the Reverse Transcription System (Promega). mRNA expression was determined by qPCR using GoTaq^®^ qPCR Master Mix (Promega) under standard thermocycler conditions (AG 22331; Eppendorf, Hamburg, Germany).

The primers used were as follows: CFL1 forward, 5′-TGTGGCTGTCTCTGATGGAG-3′ and reverse, 5′-TTGTCTGGCAGCATCTTGAC-3′; GAPDH forward, 5′-GTTCCAGTATGACTCTACCC-3′ and reverse, 5′-AGTCTTCTGAGGCAGTGATG-3′.

The following experimental run protocol was used: Denaturation program, 95°C for 1 min; and an amplification and quantification program, 45 cycles of 95°C for 45 sec, 58°C for 45 sec, 72°C for 45 sec with final fluorescence measurement. Inhibition was evaluated by quadruplication assay.

The inhibitory effect was measured using the following formula: Relative gene expression value = 2^-ΔΔCt^; ΔCt = Ct_CFL1_ - Ct_GAPDH_; ΔΔCt = ΔCt_experimental group_ - ΔCt_control group_.

### Statistical analysis

Statistical analysis was performed using SPSS 13.0 software (SPSS, Inc., Chicago, IL, USA). Differences were analyzed using Student’s t-test and the Mann-Whitney U test. Values are presented as the mean ± standard deviation. P<0.05 was considered to indicate a statistically significant difference between values.

## Results

### Establishment of RR-U251 cells

**Figure 1:**
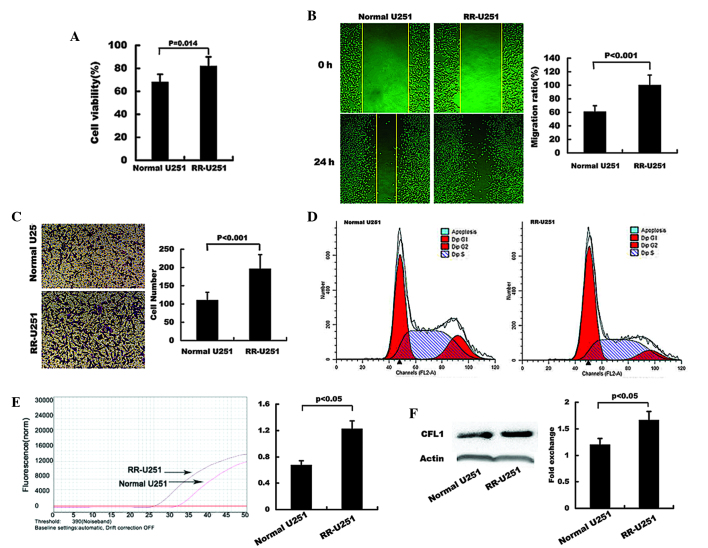
Establishment of RR-U251 cells. Following radiotherapy, (A) cell viability, (B) cell migration and (C) invasion ability were evaluated in normal U251 and RR-U251 cells. (D) Cell cycle analysis was performed in normal U251 and RR-U251 cells. (E) Reverse transcription quantitative polymerase chain reaction was used to determine the mRNA expression levels of CFL1 in normal U251 and RR-U251 cells. Results are presented as the fold increase relative to human GAPDH. (F) Western blot analysis was used to detect protein expression levels of CFL1 in normal U251 and RR-U251 cells. β-actin served as the loading control and the relative expression of CFL1 was determined using densitometry. Data are representative of three independent experiments. RR, radioresistant; CFL1, cofilin-1; mRNA, messenger RNA.

RR-U251 cells were established from normal U251 cells irradiated using a ^60^Co source at 0.5 Gy/min for 10 min per exposure until the accumulated exposure was 60 Gy. Radiosensitivity was characterized by measuring cell viability, cell cycle distribution as well as migration and invasion abilities following radiotherapy. The results showed that the cell viability, migration and invasion were significantly increased in RR-U251 cells compared with those of the normal U251 cells (Fig. 1A–C, respectively). Following radiotherapy, the percentage of cells arrested in G2 phase was 16.20% in U251 cells, compared with 8.44% in RR-U251 cells (Fig. 1D); this therefore suggested that radiosensitivity was decreased in RR-U251 cells. Elevated mRNA and protein expression levels of CFL1 were observed in RR-U251 cells compared with those of normal U251 cells (Fig. 1E and F, respectively).

### Establishment of CFL1-silenced U251 cells and CFL1-overexpressing U251 cells

**Figure 2:**
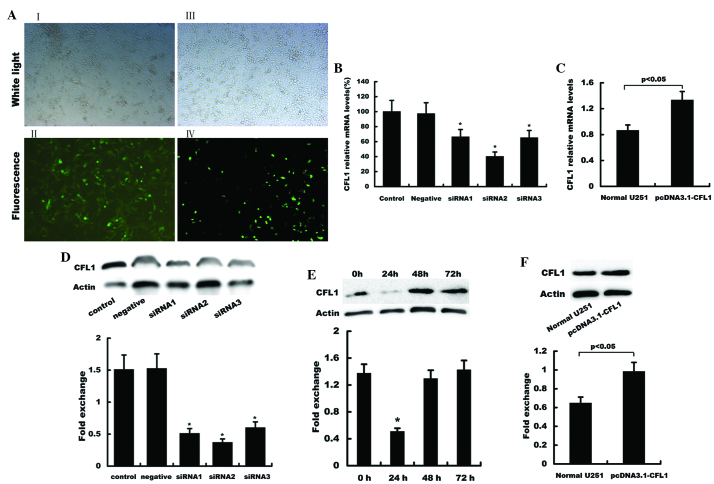
Establishment of CFL1-silenced and CFL1-overexpressing U251 cells. (A) Cells were transfected with (I, II) green fluorescent protein-siRNA or (III, IV) pcDNA3.1-CFL1 using Lipofectamine™ 2000. Following 24 h of transfection, plates were observed under bright field and fluorescence microscope systems. Reverse transcription quantitative polymerase chain reaction was used to evaluate the mRNA expression of CFL1 in U251 cells following transfection with (B) siRNA1, siRNA2 and siRNA3 as well as (C) pcDNA3.1-CFL1. Results are presented as the fold increase relative to the expression of human GAPDH, determined using densitometric analysis (n=3). Western blot analysis was used to detect protein expression of CFL1 in U251 cells following transfection with (D) siRNA1, siRNA2 and siRNA3, (E) siRNA2 for 24, 48 and 72 h, as well as (F) pcDNA3.1-CFL1. β-actin served as the loading control. CFL1, cofilin-1; siRNA; small interfering RNA; mRNA, messengerRNA.

Transfection of siRNA-CFL1 duplexes led to stable exogenous gene expression in U251 cells, with ~85–90% efficiency as indicated by the green fluorescent protein reporter (Fig. 2AI, II). Compared with those of the control group, all three duplexes significantly inhibited CFL1 mRNA and protein expression (Fig. 2B and D, respectively). Of note, siRNA2 had a more potent silencing effect compared with that of siRNA1 and siRNA3. As shown in Fig. 2E, CFL1 protein expression was significantly silenced at 24 h following transfection compared with that of the 0, 48 and 72 h groups; therefore, CFL1-siRNA2 transfected for 24 h was used for all subsequent experiments.

Transfection with pcDNA3.1-CFL1 led to a stable exogenous gene expression in U251 cells with ~30–40% efficiency (Fig. 2AIII, IV). Three weeks following G418 selection for stable clones, mRNA and protein expression levels of CFL1 were found to be significantly upregulated in U251 cells treated with pcDNA3.1-CFL1, as confirmed by RT-qPCR and western blot analysis (Fig. 2C and F, respectively).

### Radiotherapy and cell viability

**Figure 3:**
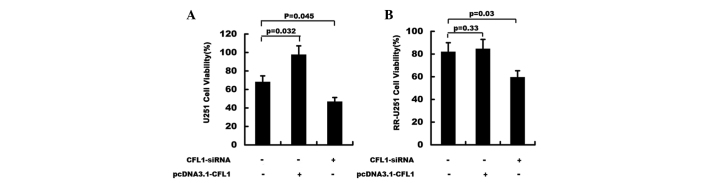
Effects of CFL1 on the proliferation of U251 and RR-U251 cells. (A) Normal U251 cells and (B) RR-U251 cells transfected with CFL1-siRNA or pcDNA3.1-CFL1 and untreated cells were exposed to a single dose of ^60^Co. The proliferation ratio was the determined using an MTT assay following 48 h (n=3). CFL1, cofilin-1; RR, radioresistant; siRNA; small interfering RNA.

Following radiotherapy, the cell viability of U251 cells was assessed using the MTT method. As shown in Fig. 3, cell viability was significantly decreased in CFL1-silenced U251 and CFL1-silenced RR-U251 cells compared to that of the control groups; in addition, overexpression of CFL1 through transfection of pcDNA3.1-CFL1 resulted in significantly enhanced proliferation in normal U251 cells (Fig. 3). These results indicated that downregulation of CFL1 may significantly elevate the radiosensitivity of U251 and RR-U251 cells.

### Radiotherapy and cell cycle distribution

**Figure 4:**
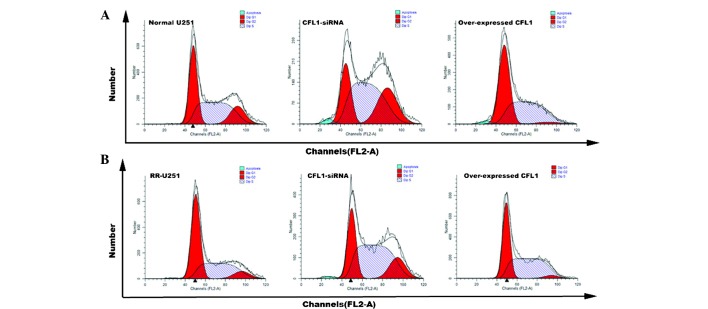
Cell cycle analysis of CFL1-silenced and CFL1-overexpressing U251 cells and RR-U251 cells. (A) Normal U251 cells and (B) RR-U251 cells transfected with CFL1-siRNA or pcDNA3.1-CFL1 and untreated cells were exposed to a single dose of ^60^Co. Flow cytometry was then performed for cell cycle analysis. CFL1, cofilin-1; RR, radioresistant; siRNA; small interfering RNA.

Flow cytometry was used to analyze cell cycle distribution following radiotherapy. Compared with that of the control groups, the number of cells arrested in G2 phase was significantly increased in normal U251 and RR-U251 cells transfected with CFL1-siRNA (Fig. 4); in addition, the number of cells arrested in G2 phase was significantly decreased in normal U251 and RR-U251 cells transfected with pcDNA3.1-CFL1. These results demonstrated that CFL1 expression affected the cell cycle in human U251 cells following radiotherapy. Cells arrested in G2 phase may be prone to apoptosis; therefore, the reduction of CFL1 may increase the number of apoptotic cells as well as increase radiosensitivity.

### Radiotherapy and cell migration ability

**Figure 5:**
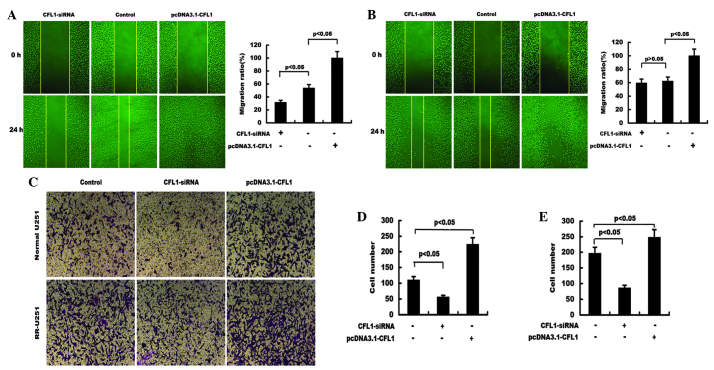
Effect of CFL1 on U251 cell migration and invasion ability. (A) Normal U251 cells and (B) RR-U251 cells transfected with CFL1-siRNA or pcDNA3.1-CFL1 and untreated cells were exposed to one dose of ^60^Co. Monolayer cells were scratched using the tip of a 10-μl pipette to create the wound line. Images were captured at 0 and 24 h at identical sites using a fluorescence microscope. Quantified results are shown in the corresponding graphs. (C) Normal U251 and RR-U251 cells transfected with CFL1-siRNA or pcDNA3.1-CFL1 and untreated cells were exposed to a single dose of ^60^Co. Cell invasion ability was evaluated using a Transwell culture chamber system. Bar graphs show the number of invading (D) U251 cells and (E) RR-U251 cells (n=3). CFL1, cofilin-1; RR, radioresistant; siRNA; small interfering RNA.

The migration ability of untreated cells and cells treated with CFL1-siRNA or pcDNA3.1-CFL1 were examined using a wound healing assay. As shown in Fig. 5A and B, following radiotherapy, CFL1-silenced U251 cells showed significantly decreased migration ability compared with that of the control cells. By contrast, CFL1-overexpressing cells showed significantly enhanced migration abilities in normal U251 and RR-U251 cells. These results indicated that downregulation of CFL1 significantly reduced the migration ability of U251 cells and elevated the radiosensitivity of normal U251 and RR-U251 cells.

### Radiotherapy and cell invasion ability

Cell invasion ability was determined using a Transwell chamber system. Compared with that of the control, the invasion potential of U251 cells transfected with CFL1-siRNA was significantly decreased in normal U251 and RR-U251 cells, whereas cells transfected with pcDNA3.1-CFL1 demonstrated markedly increased invasive abilities (Fig. 5C–E). These results indicated that downregulation of CFL1 significantly reduced the invasion ability of U251 cells and elevated the radiosensitivity of normal U251 and RR-U251 cells.

## Discussion

Human intracranial glioma is the most common type of primary malignant tumor; it is highly invasive and has unclear boundaries with surrounding tissues (15). Six months following surgery, infiltrative tumor cells may invade other issues, rapidly resulting in glioma recurrence (16). Surgery is the preferred treatment for glioma, and is combined with chemotherapy or radiotherapy in order to eradicate the tumor metastatic small lesions. Compared with chemotherapy, radiotherapy is a more effective treatment for conformal therapy to the target irregular sections without the limits of the blood-brain barrier (17); therefore, it has become the most important treatment for malignant glioma following surgery.

However, numerous factors have been shown to restrict the effects of radiotherapy. Previous studies have suggested that radioresistance may be caused by interactions between tumors and their microenvironment through angiogenesis (18), hypoxia (19) and immunosuppressive processes (20). Conversely, other studies have shown that radiotherapy may induce cell cycle arrest, DNA repair and apoptosis (21), therefore indicating that these factors critical for radiosensitivity were the result of interactions between intracellular proteins or genes.

A previous study reported that CFL1 was significantly upregulated in radioresistant astrocytomas (2); these findings suggested that CFL1 may be correlated with radiosensitivity in glioma. CFL1, an actin-binding protein, has a critical role in the cell cytoskeleton maintaining cellular homeostasis and participates in numerous physiological activities (22). Studies have shown that cofilin was a critical factor for tumor metastasis and drug resistance to chemotherapy (23–25). Cofilin acts as an important regulatory factor in tumor cell invasion and metastasis via the formation of lamellipodia, which therefore promote cell migration (26). Castro *et al* (27) identified CFL1 as a potential biomarker for the prognosis of non-small cell lung cancer, where it was found to be associated with resistance to alkylating drugs. In addition, CFL1 was reported to be highly expressed in highly invasive cells (28–31), including breast cancer, colon cancer and malignant glioma cells. These previous studies therefore indicated that cofilin was essential for tumor progression, cell motility, cell adhesion, cell invasion and angiogenesis.

Studies have shown that the extent of malignancy and recurrence of gliomas was associated with cell motility. CFL1, a key protein in cell movement, may promote the formation of filopodia and enhanced cell motility (32). CFL1 was reported to be overexpressed in cells with high metastatic and invasion abilities, including hepatoma carcinoma, breast cancer and colon carcinoma cells. The results of the present study showed that CFL1 was overexpressed in RR-U251 cells, and that the migration and invasion abilities of these cells were significantly enhanced. Furthermore, these results indicated that CFL1 overexpression decreased radiosensitivity via increasing the metastasis and invasiveness of U251 cells.

Cell cycle arrest, DNA repair and apoptosis induced by radiotherapy are the key factors which contribute to radiosensitivity (33). The results of the present study demonstrated that the number of cells arrested in G2 phase was significantly reduced in RR-U251 and CFL1-overexpressing U251 cells. This therefore indicated that the number of apoptotic cells declined and the radiosensitivity of U251 cells with high CFL1 expression decreased. In addition, CFL1-silencing in U251 cells resulted in increased radiosensitivity. These results suggested that the regulation of CFL1 in tumor cells may occur due to the priming of cell transformation, reinforcement of cell mobility in cell metastasis and the division of tumor cells. Cofilin and Lim kinase, its regulatory protein, have been shown to have critical roles in cell motility (34). The results of the present study indicated that downregulation of CFL1 may increase the radiosensitivity of U251 cells through reducing cellular migration and invasion abilities.

In conclusion, the results of the present study demonstrated that downregulation of CFL1 may increase radiosensitivity in U251 cells *in vitro*; however, further studies are required in order to elucidate the exact molecular mechanism of this. In addition, further studies are required in order to determine the role of CFL1 *in vivo*.
